# Continental drift and climate change drive instability in insect assemblages

**DOI:** 10.1038/srep11343

**Published:** 2015-06-17

**Authors:** Fengqing Li, José Manuel Tierno de Figueroa, Sovan Lek, Young-Seuk Park

**Affiliations:** 1 Department of Biology, Kyung Hee University, Seoul 130-701, Republic of Korea; 2 Department of Zoology, Faculty of Sciences, University of Granada, Granada 18071, Spain; 3 Lab EDB (Evolution & Diversité Biologique), UMR CNRS-Université Paul Sabatier, Université de Toulouse, Toulouse 31062, France; 4 Department of Life and Nanopharmaceutical Sciences, Kyung Hee University, Seoul 130-701, Republic of Korea

## Abstract

Global change has already had observable effects on ecosystems worldwide, and the accelerated rate of global change is predicted in the future. However, the impacts of global change on the stability of biodiversity have not been systematically studied in terms of both large spatial (continental drift) and temporal (from the last inter-glacial period to the next century) scales. Therefore, we analyzed the current geographical distribution pattern of Plecoptera, a thermally sensitive insect group, and evaluated its stability when coping with global change across both space and time throughout the Mediterranean region—one of the first 25 global biodiversity hotspots. Regional biodiversity of Plecoptera reflected the geography in both the historical movements of continents and the current environmental conditions in the western Mediterranean region. The similarity of Plecoptera assemblages between areas in this region indicated that the uplift of new land and continental drift were the primary determinants of the stability of regional biodiversity. Our results revealed that climate change caused the biodiversity of Plecoptera to slowly diminish in the past and will cause remarkably accelerated biodiversity loss in the future. These findings support the theory that climate change has had its greatest impact on biodiversity over a long temporal scale.

Global biodiversity is undergoing extensive change at an unprecedented rate due to the combined forces of ecological, environmental, evolutionary, and historical stressors^1^,^2^,^3^. However, the impacts of global stressors on the stability of insect diversity are still largely unclear because their effects are generally acknowledged to be species-specific, and detailed natural history information is not available for most insect species^4^,^5^,^6^. Nevertheless, using a regional, well-studied insect group makes it possible to evaluate the stability of biodiversity in the face of one or more global stressors (i.e., global change). The Plecoptera (also called stonefly) is one such group.

Plecoptera is a minor order of insect fauna but plays important roles in the running waters^7^. Plecoptera is widely distributed over all continents except Antarctica, and currently, more than 3,497 species are described^8^. As an ancient insect group, the extant Plecoptera assemblages provide insights into geographical history. For example, the breakup of Pangaea into Laurasia and Gondwanaland seems to have caused the separation of Plecoptera into distinct southern and northern hemisphere suborders, i.e., Antarctoperlaria and Arctoperlaria^9^. Plecopteran nymphs mainly inhabit cold and clean running waters, and their basic physiological functions are extensively influenced by external thermal conditions^9^,^10^,^11^,^12^. These characteristics, in addition to their low dispersal capacity, make Plecoptera an excellent animal model with which to detect global change^13^.

To cope with global change, organisms can shift their distributional ranges to track more favorable habitats (i.e., the ecological strategy), or persist in their original habitats through phenotypic plasticity or rapid evolutionary adaptation (i.e., the genetic strategy)^14^,^15^,^16^,^17^. Extinction will occur if organisms fail to adapt to new environmental condition by means of either ecological or genetic strategy^5^,^12^,^18^,^19^,^20^. Although rapid evolutionary adaptation is possible, movement to track favorable habitats is generally acknowledged as the more common response of organisms^21^. Therefore, we hypothesize that range shifts and loss of biodiversity causes the instability of the Plecoptera assemblage at both large spatial (continental drift) and temporal (from the last inter-glacial to the next century) scales. To test our hypothesis, we selected one of the first 25 global biodiversity hotspots—the Palearctic Mediterranean region— as our study region because of its sensitive climatic conditions and representative insect diversity^22^. This region lies in a transition zone between the subtropical arid climate of North Africa and the temperate climate of South Europe. Given this location, even minor changes in atmospheric dynamics can lead to large variations in climate (a so-called “climatic sensitive” region)^23^. These large variations in climate produce diverse plant communities, food resources, and habitats for insects, which results in an unusually high Plecoptera richness in the Mediterranean region when compared with other regions also at 40° N latitude^24^. In addition, high Plecoptera richness is found in the Mediterranean region because it is one of the most important glacial refuges for Plecoptera assemblages^11^.

In our study, we compiled survey data of Plecoptera from the western Mediterranean region. We then extracted 18 sub-regions from these data to evaluate the stabilities of Plecoptera assemblages in the context of the effects of land uplift (continental drift) and long-term climatic variations (from the last inter-glacial [LIG, 120–140 thousand years (Ka) ago] to the future 100 years) (Supplementary Fig. S1). Based on the differences in Plecoptera diversity at both spatial and temporal scales, we particularly aimed to test the following two hypotheses: (1) in contrast to the broadly acknowledged biogeographical isolation theory, at the borders of continents there is less similarity of Plecoptera assemblages within rather than between continents (i.e., Africa and Europe) due to the effect of continental drift, and (2) Plecoptera richness is higher in the last glacial maximum than in the last inter-glacial because plecopteran nymphs prefer low temperatures.

## Results

### Biodiversity across space

**Figure 1:**
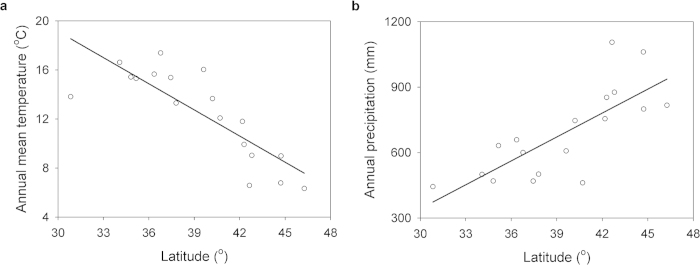
Latitudinal gradients of (**a**) annual mean temperature and (**b**) annual precipitation in the western Mediterranean region between 1950 and 2000.

Patterns of climatic variables indicated that from the subtropical arid climate of North Africa to the rainy temperate climate of South Europe, temperature decreased (*F*_*1*,*16*_ = 33.68, *R*^*2*^ = 0.68, *P* < 0.01; Fig. 1a) and precipitation increased (*F*_*1*,*16*_ = 22.01, *R*^*2*^ = 0.58, *P* < 0.01; Fig. 1b) along the latitudinal gradient. However, annual mean temperature and annual precipitation were not significantly related to longitude (Supplementary Fig. S2a, b), but they were related to elevation (temperature: *F*_*1*_,_*16*_ = 24.91, *R*^*2*^ = 0.61, *P* < 0.01; precipitation: *F*_*1*,*16*_ = 4.52, *R*^*2*^ = 0.22, *P* = 0.05; Supplementary Fig. S2c, d).

**Figure 2:**
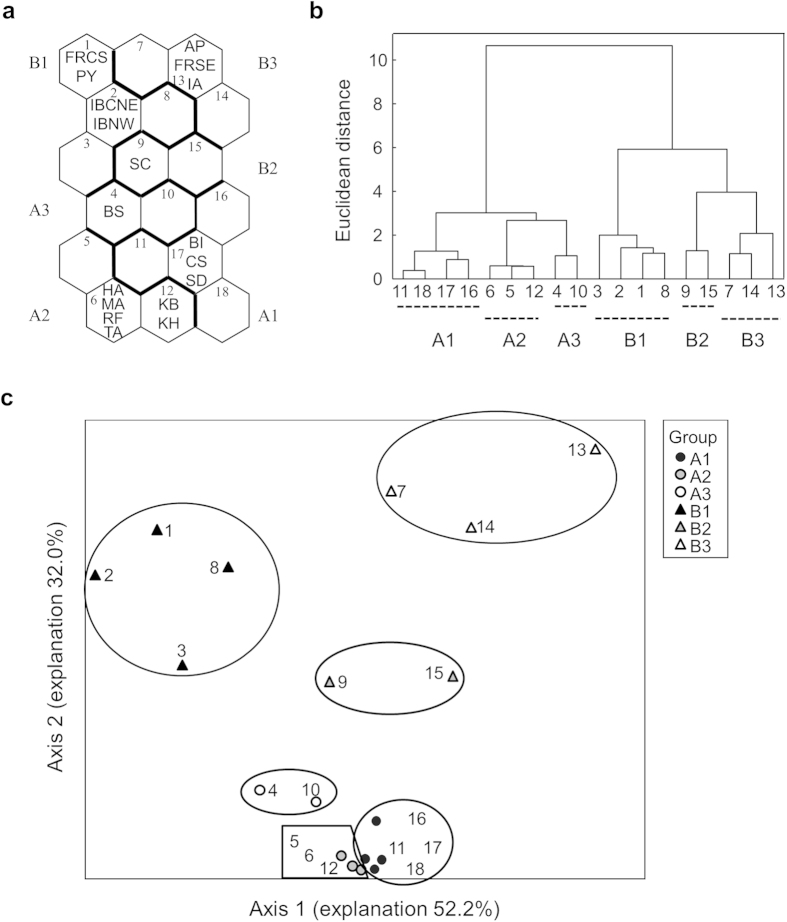
Classification of the sub-regions defined by the self-organizing map (SOM). (**a**) Ordination of sub-regions based on SOM, (**b**) cluster analysis of the SOM units using Ward’s linkage method, and (**c**) distributional pattern of 18 SOM units defined by nonmetric multidimensional scaling (NMDS). The full name of each sub-region is provided in Supplementary Table S1.

A similar climatic pattern structured the biodiversity map in the western Mediterranean region. We differentiated these biogeographical patterns using a self-organizing map (SOM), which utilizes efficient machine learning algorithms. In total, all 18 sub-regions of the study area were classified into six groups (Fig. 2a,b). Sub-regions from France, Italy, and Spain (except the Baetic System) were mainly in the upper area of the map, and these sub-regions together with Sicily (in the central upper area of the map) were clustered in group B. Sub-regions from the bottom of SOM belonged to group A, including the Baetic System, the larger Mediterranean islands, and North Africa (Fig. 2a,c).

The occurrence and richness of Plecoptera were lower in North Africa (occurrence: 36, richness: 18) and on islands (48, 22), but higher in South Europe (271, 87; Supplementary Fig. S3). A significant increase in Plecoptera richness was observed along the latitudinal (*F*_*1*,*16*_ = 18.41, *R*^*2*^ = 0.54, *P* < 0.01; Supplementary Fig. S4a, b) and altitudinal gradients (*F*_*1*,*16*_ = 14.19, *R*^*2*^ = 0.47, *P* < 0.01; Supplementary Fig. S4b). However, there was no significant trend along the longitudinal gradient (Supplementary Fig. S4a).

### Biodiversity across time

**Figure 3:**
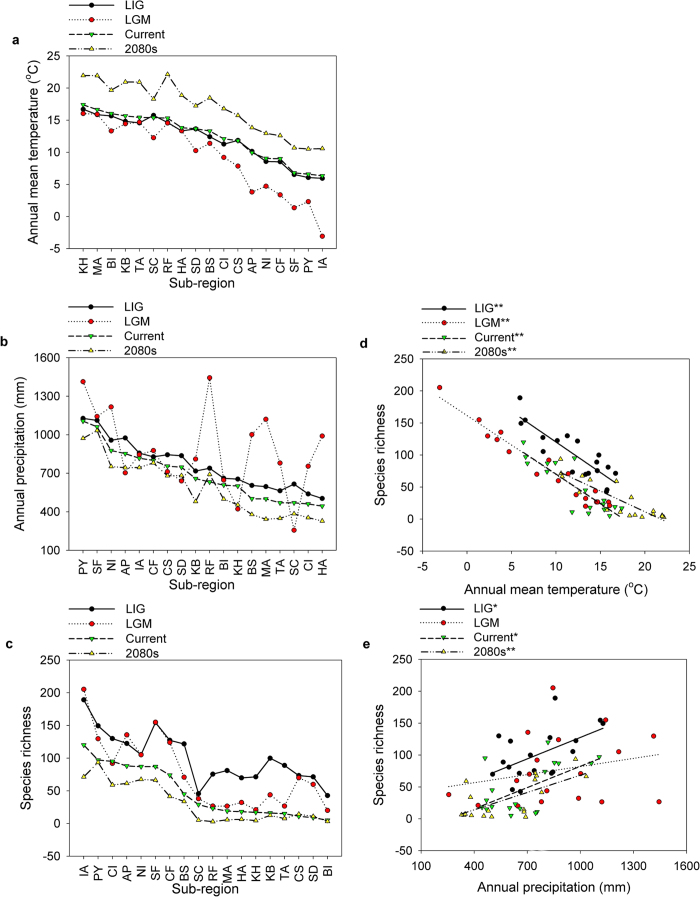
Rank of (**a**) annual mean temperature, (**b**) annual precipitation, and (**c**) predicted Plecoptera richness in four climatic periods (the last inter-glacial [LIG], the last glacial maximum [LGM], current, and the 2080s), and the relationships between the predicted Plecoptera richness and (**d**) temperature and (**e**) precipitation. The full name of each sub-region is provided in Supplementary Table S1. Simple linear regressions are given in (**d**) and (**e**). ** *P* < 0.01 and * *P *< 0.05.

Temporal trends showed that the annual mean temperature in the current climatic period was similar to that of the two paleoclimatic periods (i.e., the last inter-glacial and last glacial maximum) but was significantly lower than that of the 2080 s (paired *t*-test: *t* = 3.40, *P* < 0.01; Fig. 3a). Annual precipitation patterns were similar among all periods except for last glacial maximum (paired *t*-test between the current period and the last glacial maximum: *t* = 2.15, *P* = 0.04; Fig. 3b). The predicted Plecoptera richness was significantly higher in last inter-glacial than in the current period (paired *t*-test: *t* = 4.10, *P* < 0.01; Fig. 3c) and in the 2080 s (paired *t*-test: *t* = 5.92, *P* < 0.01; Fig. 3c), but there was no significant difference between the last inter-glacial and last glacial maximum (paired *t*-test: *t* = 1.53, *P* = 0.14; Fig. 3c).

**Figure 4:**
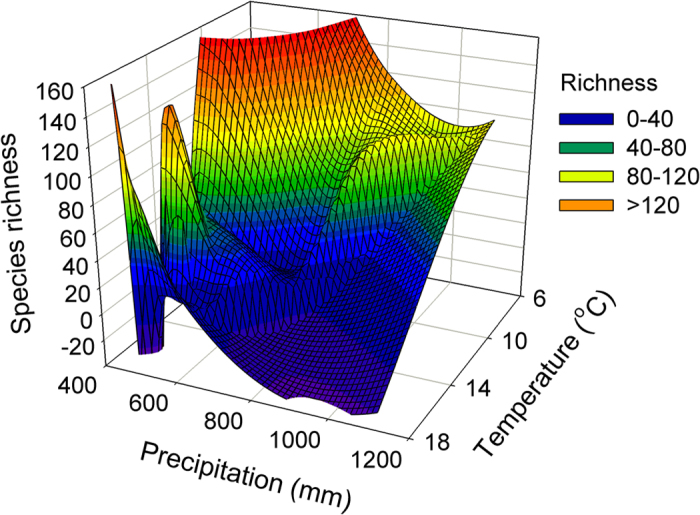
Plecoptera richness in the current climatic period along temperature and precipitation gradients in the western Mediterranean region.

Plecoptera richness was not only significantly related to temperature in all periods (*R*^*2*^: 0.75−0.95, *P* < 0.01; Fig. 3d) but was related to precipitation in some periods (*R*^*2*^: 0.06−0.44, *P*: 0.01−0.33; Fig. 3e). Specifically, Plecoptera richness decreased significantly along the temperature gradient (*F*_*1, 16*_ = 50.67, *R*^*2*^ = 0.76, *P* < 0.01; Fig. 4) but increased along the precipitation gradient (*F*_*1, 16*_ = 8.34, *R*^*2*^ = 0.34, *P* = 0.01; Fig. 4).

## Discussion

Regional biodiversity of Plecoptera well reflected the geographical pattern in both historical movements of continent and the current distribution condition in the western Mediterranean region. We are also aware that climate change is not only a matter of the future^14^,^15^,^16^,^17^, as our results strongly support the theory that climate change has had its greatest impact on biodiversity over a large past temporal scale.

From the subtropical arid climate in North Africa to the rainy temperate climate in South Europe, the temperature decreased and the precipitation increased along the latitudinal gradient (Fig. 1). Such climatic pattern also structured the biodiversity map in the western Mediterranean region. From the regional biodiversity point of view, the community similarity of Plecoptera between the southern border of Europe (i.e., the Baetic System) and North Africa is higher than that between the southern border of Europe and the European continent, which is in agreement with our first hypothesis. However, this finding contrasts with the widely accepted biogeographical isolation theory. In this theory, the similarity between communities is generally low if the geographical isolation works (e.g., by the Mediterranean Sea, in our case)^25^. One explanation for this inconsistency is continental drift. Paleogeographical maps show that the Baetic System was visible since the Oligocene (25 million years, Ma). The Baetic System continued to uplift and enlarge throughout the Miocene (13 Ma) until the northern section became part of the European continent, the southern section became part of Africa, and the remaining sections became islands in the Mediterranean Sea (Supplementary Fig. S5). These movements are corroborated by the fact that drifting ice in western Europe carried soil and associated biota to parts of Scandinavia, Iceland, and Greenland during the post-Pleistocene (2.5 Ma)^26^. Additional support is also provided by the fauna similarities between eastern South America and western Central Africa, between Andean South America and Australia, between eastern North America and Europe, and between Russian Far East and northwestern North America^27^.

Insects are an ancient taxon, and most orders of insects are older than many birds and mammals^9^. Particularly, Plecoptera existed at least since the Permian, and the oldest known fossils date back to 258−263 Ma^28^. Nevertheless, recently a stem-stonefly has been reported from the Pennsylvanian, extending the fossil record into the Carboniferous, about 300 million years ago^29^. Therefore, the currently disjunct distribution of this monophyletic taxon can be an excellent indicator of continental drift and land uplift. However, continental drift alone probably cannot entirely explain the present distribution of Plecoptera. Ecological factors (e.g., climate change, dispersal capacity, and species interactions) remain important^30^. Ecological change, perhaps past warming and drying, caused a considerably low Plecoptera richness in North Africa. Based on this assumption, we further evaluated the temporal stability of Plecoptera assemblages from the last inter-glacial to the future 100 years (the second hypothesis).

The second hypothesis, however, was not supported by our study since there was no significant difference of the predicted Plecoptera richness between last inter-glacial and last glacial maximum (Fig. 3c). As identified by Brittain^4^, Plecoptera is able to adapt easily to low temperature, but high temperatures cause a decrease in egg hatching rate and nymph survival rate and further affect plecopteran development due to the lower oxygen level^10^. The temperature limit for most species of Plecoptera seems to be approximately 25 °C, and this limit can be considerably lower for some species^10^. Thus, it is likely that the temperature difference between the last inter-glacial and last glacial maximum was not large enough to affect Plecoptera richness in this region, although higher temperature during the last inter-glacial could probably shrink the global distribution of some species at higher altitude (particularly considering the mountainous nature of the study region) without causing a sharp decline in the diversity of Plecoptera. Nevertheless, temperature increase in the future could have primarily negative effects on biodiversity by dramatically reducing the number of available habitats (Fig. 3c)^13^.

The intermediate level of precipitation resulted in higher Plecoptera richness within the range of 720 (modeled maximum precipitation in Fig. 4) and 817 mm (the sub-region with the highest species richness). This finding is in agreement with the intermediate environmental disturbance theory^31^. In comparison to this optimal precipitation range, the mean precipitation in the last glacial maximum was relatively high (876 mm), whereas the last inter-glacial was in the optimal range (763 mm). Nevertheless, the predicted Plecoptera richness was lower in the 2080 s due to its low mean precipitation (592 mm) and high mean temperature (16.9 °C). This trend suggests that the biodiversity of Plecoptera decreased due to climate change in the past 120–140 Ka. The decline in Plecopteran biodiversity occurred slowly in the past (i.e., loss of 0.58 species per Ka since the last inter-glacial and 1.39 species per Ka since the last glacial maximum) and at remarkably high speed in the future (i.e., loss of 160 species per Ka in the next 100 years). Possible explanations for this increase in the speed of Plecopteran decline include ecological constraints (e.g., dispersal capacity) and physical limits or geographical isolation (e.g., due to high mountains, deserts, or oceans). Most Plecoptera are poor dispersers; for example, in an experimental study conducted in the UK, 90% stoneflies traveled less than 60 m laterally from the studied streams^32^. Physical limits such as geographical isolation can further increase habitat fragmentation and reduce population connectivity^19^.

In conclusion, the regional biodiversity of Plecoptera was found to agree with both historical continental movements and current environmental conditions in the western Mediterranean region. Our results strongly support the theory that climate change has had its greatest impact on biodiversity over a long temporal scale. Plecoptera is thus an efficient taxonomic tool with which to detect the impacts of geographical and climatic changes on the stability of regional biodiversity. Despite the relatively scarce current molecular studies on European Plecoptera do not yet allow evaluating the existence of the enormous cryptic diversity found in some other aquatic organisms^33^,^34^,^35^,^36^, we are aware that the natural diversity of Plecoptera could be larger than predicted from current taxonomical knowledge and the real distribution patterns could be slightly different, and this could lead to potential follow-up studies. Our study not only provides evidence for the profound effects of human-induced global change on regional biodiversity but also illustrates a more complete picture of biodiversity stability in the context of both spatial and temporal scales. The high rate of species loss, currently thought to be 0.58 to 160 species per Ka, may be much stronger than previously thought. To stabilize regional biodiversity and mitigate the influences of global change, conservation management (e.g., reducing pollution, improving habitat quality, and increasing canopy cover) may help to a certain extent.

## Methods

### Data collection

We compiled a set of geographical qualitative records (i.e., presence/absence data) of Plecoptera across the western Mediterranean region, including North Africa, South Europe, and several large islands in the Mediterranean Sea. Data of France were obtained from the Protection of Insect Environment (http://www.opie-benthos.fr), and all other data were obtained from the University of Granada in Spain. In total, 306 species of Plecoptera were observed in the western Mediterranean region. Taxa of Plecoptera observed in each sub-region is available in Supplementary Table S2.

We extracted 18 sub-regions from the study region according to the similarity of geographical characters (Supplementary Table S1, Fig. S1). The geographical coordinates of each sub-region were the approximate geographical center and a buffer zone with a diameter of 50 km was used to extract the average elevation and climatic variables in each sub-region. Digital elevation maps were obtained from the U.S. National Aeronautics and Space Administration (https://wist.echo.nasa.gov; resolution: 30 arc-seconds). Six climatic variables that affect energy and water regimes were used to calibrate the species distribution models^37^,^38^: annual mean temperature, mean temperature of the warmest quarter, mean temperature of the coldest quarter, annual precipitation, precipitation of the warmest quarter, and precipitation of the coldest quarter. We used four climatic periods (the paleoclimatic last inter-glacial [120–140 Ka], the paleoclimatic last glacial maximum [21 Ka], the current period [1950–2000], and the future 100 years [the 2080s, averaged from 2070–2099]) to test the temporal trends of climatic and biotic variables. The climatic variables of the paleoclimatic periods (resolution: 30 arc-seconds and 2.5 arc-minutes for the last inter-glacial and the last glacial maximum, respectively) and the current period (30 arc-seconds) were obtained from WorldClim (http://www.worldclim.org). Climatic variables for the 2080s were obtained from the CIAT database (http://www.ccafs-climate.org; resolution: 30 arc-seconds) based on A1B emission scenario (720 ppm atmospheric CO_2_ in the 2100s). We selected the A1B emission scenario because it includes a balanced mix of all energy sources and closely matches the most likely future status of national energy sources^12^,^39^. The paleogeographical maps of North Africa, Europe, and the Mediterranean Sea were obtained from Colorado Plateau Geosystems, Inc. (http://cpgeosystems.com). Three paleoclimatic periods were incorporated: Eocene (50 million years, Ma), Oligocene (25 Ma), and Miocene (13 Ma).

### Statistics

To test our first hypothesis that the similarity of Plecoptera assemblages is lower within a continent rather than between continents (i.e., North Africa and South Europe), two approaches were used to visualize the distributional pattern of sub-regions: self-organizing map (SOM) and nonmetric multidimensional scaling (NMDS). SOM consists of input and output layers connected by connection intensities. The input layer obtains information from the data matrix, while the output layer visualizes the computational results by using a two-dimensional hexagonal lattice^40^,^41^. The output nodes are considered to be virtual units that represent the typical pattern of the input data matrix assigned to their units after the learning process. We used 18 ( = 6 × 3) output nodes to pattern the sub-regions. The map size of the output nodes was chosen according to a heuristic equation 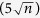
42. After the learning process of SOM, the SOM units were classified into groups based on the dendrogram of a hierarchical cluster analysis with Ward’s linkage method using Euclidian distance^40^,^41^. The above process was performed by the SOM toolbox in Matlab (version 6.1)^43^. NMDS was performed using PC-ORD version 5.3. A two dimensional solution was used for all analyses in deriving stress values. A Monte Carlo test with 50 iterations was used to evaluate whether NMDS extracted stronger axes than expected by chance.

To test our second hypothesis that the Plecoptera richness is higher in the last glacial maximum than in the last inter-glacial, a generalized additive model (GAM) was used to predict the Plecoptera richness in the last inter-glacial, last glacial maximum, and the 2080s^2^,^44^. GAM quantifies the relationships between six climatic variables and Plecoptera richness in the current period. The results of this model are used to estimate Plecoptera richness in paleoclimatic and future climatic periods by comparing the climatic variations between the current and predicted periods^2^. This formula is shown in eq. (1):


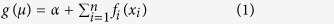


where *g*(*μ*) is the response (i.e., the predicted species richness), *f*_*i*_ is the smoothing function, *x*_*i*_ is the explanatory variables (i.e., six climatic variables) at site *i*, *n* is the number of explanatory variables and *a* is the residual error.

GAMs were conducted by using the *gam* package in R (R project, Vienna, Austria; http://cran.r-project.org). Paired *t*-tes*t*s were used to determine the difference between the climatic variables and predicted Plecoptera richness for each of the four climatic periods. Paired *t*-tes*t*s were also conducted in R.

## Additional Information

**How to cite this article**: Li, F. *et al*. Continental drift and climate change drive instability in insect assemblages. *Sci. Rep*. **5**, 11343; doi: 10.1038/srep11343 (2015).

## Supplementary Material

Supplementary Information
