# Supplementary material for: Continental drift and climate change drive instability in insect assemblages
Source: Sci Rep. 2015 Jun 17;5:11343. doi: 10.1038/srep11343 (PMC4469969; doi:10.1038/srep11343)
Supplement: Supplementary Information [file srep11343-s1.pdf]

Supplementary Information

Continental drift and climate change drive instability in insect assemblages

Fengqing Li[1], José Manuel Tierno de Figueroa[2], Sovan Lek[3], and Young-Seuk Park[1,4*]

[1] Department of Biology, Kyung Hee University, Seoul 130-701, Republic of Korea

[2] Department of Zoology, Faculty of Sciences, University of Granada, Granada 18071, Spain

[3] Department of Biology, Université de Toulouse, Toulouse 31062, France

[4] Department of Life and Nanopharmaceutical Sciences, Kyung Hee University, Seoul 130-701, Republic of Korea

[*]Corresponding author: Y.-S.P. (parkys@khu.ac.kr)

Table S1 Geographical information of each sub-region in the western Mediterranean region. Directionality refers to the direction of river flows; latitude and longitude are provided for the geographical center of the sub-region; and elevation is averaged from a buffer zone with a diameter of 50 km.

Sub-region	Acronym	Country	Directionality	Latitude (°)	Longitude (°)	Elevation (m)
Haut Atlas	HA	Morocco	East-west	30.83	–8.47	1392
Moyen Atlas	MA	Morocco	East-west	34.07	–4.46	709
Rif	RF	Morocco	East-west	35.15	–5.46	479
Kabylie	KB	Algeria	South-north	36.35	3.68	641
Tafna Algérois	TA	Algeria	South-north	34.81	–1.42	780
Khoumirie	KH	Tunisia	South-north	36.77	9.45	223
Balearic Islands	BI	Spain	Center-quarter	39.61	3.00	151
Baetic System	BS	Spain	East-west	37.80	–2.75	1059
Central+Northeast Iberian Peninsula	CI	Spain	East-west	40.70	–3.20	869
Northwest Iberian Peninsula	NI	Spain	North-south	42.80	–6.30	1164
Pyrenees	PY	Spain/France	Center-quarter	42.64	1.17	1497
Corsica	CS	France	Center-quarter	42.18	9.09	739
Central South France	CF	France	South-north	44.72	2.95	901
Southeast France	SF	France	South-north	44.71	5.84	1223
Apennines	AP	Italia	Center-quarter	42.29	13.47	1030
Italian Alps	IA	Italia	North-south	46.26	11.08	1270
Sicily	SC	Italia	North-south	37.45	14.41	429
Sardinia	SD	Italia	Center-quarter	40.20	9.16	585

Table S2 The number of Plecoptera species of each genus observed in each sub-region in the western Mediterranean region. The full name of each sub-region is provided in Supplementary Table S1.

Family	Genus	HA	MA	RF	KB	TA	KH	BI	BS	CI	NI	PY	CS	CF	SF	AP	IA	SC	SD
Capniidae	Capnia	2	1	1	1	0	0	0	2	3	2	3	0	3	3	3	3	1	0
Capniidae	Capnioneura	1	1	1	1	1	1	0	3	3	4	4	1	2	2	1	1	1	1
Capniidae	Capnopsis	1	0	1	1	0	1	0	1	1	0	0	0	0	0	1	0	0	0
Chloroperlidae	Chloroperla	0	0	0	0	0	0	0	1	4	4	2	0	1	2	2	2	0	0
Chloroperlidae	Siphonoperla	1	1	1	0	0	0	0	1	1	1	1	0	1	2	2	2	1	0
Chloroperlidae	Xanthoperla	0	0	0	0	0	0	0	0	1	0	1	1	1	0	1	1	0	1
Leuctridae	Leuctra	3	3	5	3	2	6	2	11	24	25	29	3	20	33	24	46	5	3
Leuctridae	Pachyleuctra	0	0	0	0	0	0	0	0	0	0	3	0	1	0	0	0	0	0
Leuctridae	Tyrrhenoleuctra	0	1	2	1	1	1	3	4	1	0	0	1	0	0	0	0	0	1
Nemouridae	Amphinemura	1	1	1	1	1	1	0	1	3	4	3	0	3	2	2	3	2	0
Nemouridae	Nemoura	0	2	2	1	0	0	0	4	7	9	12	0	10	11	11	14	4	0
Nemouridae	Nemurella	0	0	0	0	0	0	0	0	1	1	1	0	1	1	1	1	0	0
Nemouridae	Protonemura	1	2	2	2	4	2	0	3	11	13	13	2	10	11	16	15	6	1
Perlidae	Dinocras	0	0	0	0	0	0	0	1	1	1	1	0	1	2	2	3	1	0
Perlidae	Eoperla	1	1	1	1	1	1	0	1	1	0	1	0	0	0	0	0	0	0
Perlidae	Marthamea	0	0	0	0	0	0	0	1	2	1	1	0	0	0	0	0	0	0
Perlidae	Perla	2	2	1	1	2	1	0	3	5	5	3	0	3	3	3	5	2	0
Perlodidae	Afroperlodes	1	1	1	1	1	1	0	0	0	0	0	0	0	0	0	0	0	0
Perlodidae	Arcynopteryx	0	0	0	0	0	0	0	0	1	1	1	0	0	0	0	0	0	0
Perlodidae	Besdolus	0	0	0	0	0	0	0	1	1	0	1	0	1	1	1	0	0	0
Perlodidae	Dictyogenus	0	0	0	0	0	0	0	0	0	0	0	0	0	2	0	2	0	0
Perlodidae	Guadalgenus	0	0	0	0	0	0	0	0	1	0	0	0	0	0	0	0	0	0
Perlodidae	Hemimelaena	1	1	1	0	1	0	0	1	1	1	0	0	0	0	0	0	0	0
Perlodidae	Isogenus	0	0	0	0	0	0	0	0	0	0	1	0	1	0	0	1	0	0
Perlodidae	Isoperla	1	0	1	0	0	0	0	2	7	6	7	2	5	3	7	10	3	1
Perlodidae	Perlodes	0	0	0	0	0	0	0	1	3	2	3	0	2	2	2	2	0	0
Taeniopterygidae	Brachyptera	2	2	2	2	1	2	0	1	8	4	3	1	4	1	3	4	2	1
Taeniopterygidae	Rhabdiopteryx	0	0	0	0	0	0	0	1	2	2	0	0	1	4	1	2	1	0
Taeniopterygidae	Taeniopteryx	0	0	0	0	0	0	0	1	2	1	3	0	3	2	5	3	0	0

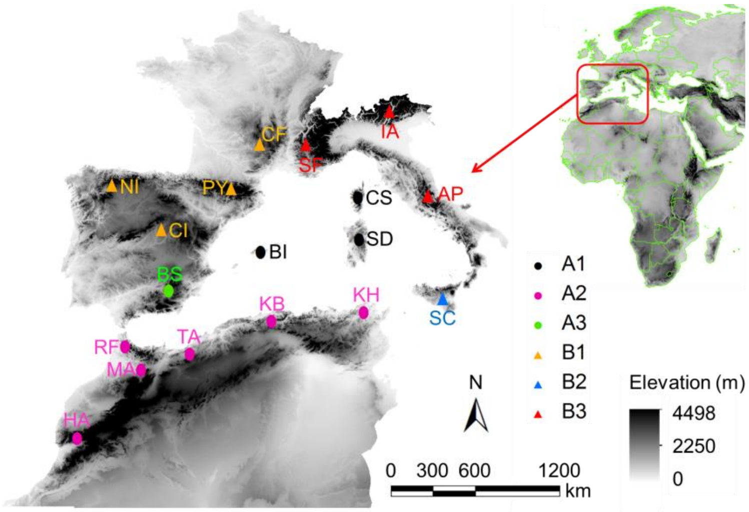

Fig. S1 Geographical locations of the sub-regions in the western Mediterranean. The full name of each sub-region is provided in Supplementary Table S1. Digital elevation map and localities of sub-regions were visualized using ArcGIS 10.2.

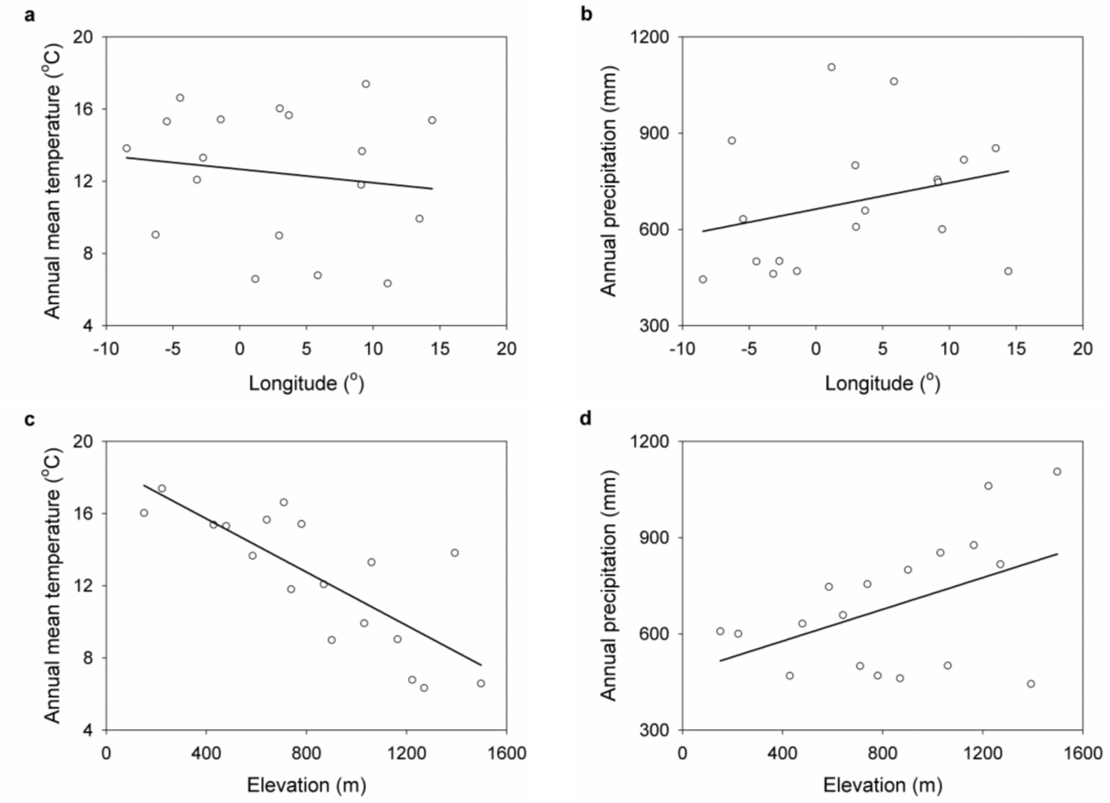

Fig. S2 Annual mean temperature and annual precipitation along the (**a**, **b**) longitudinal and (**c**, **d**) altitudinal gradients in the western Mediterranean region.

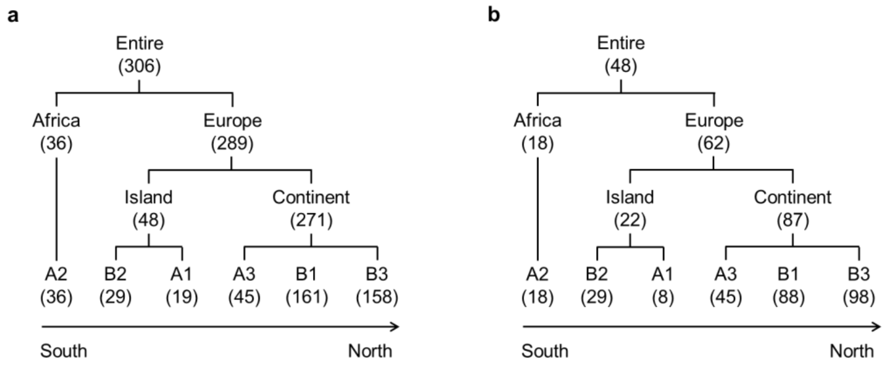

Fig. S3 (**a**) Occurrence and (**b**) average Plecoptera richness among each SOM (self-organizing map) group in the western Mediterranean region.

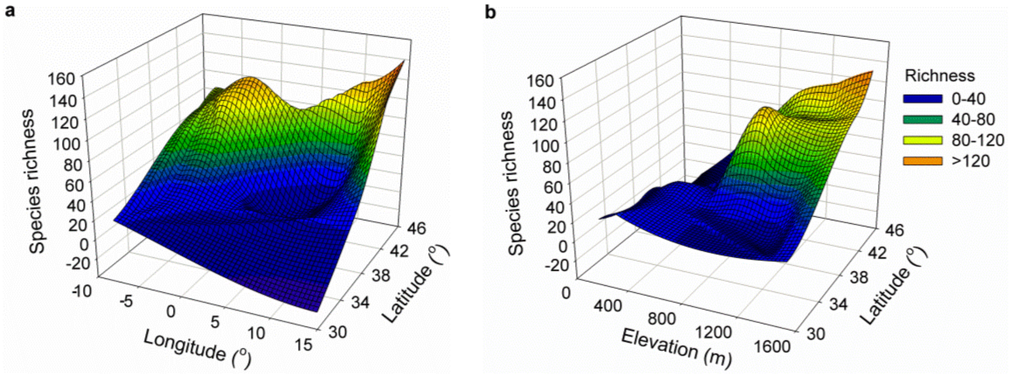

Fig. S4 Plecoptera richness in the current climatic period with respect to (**a**) latitude and longitude and (**b**) latitude and elevation in the western Mediterranean region.

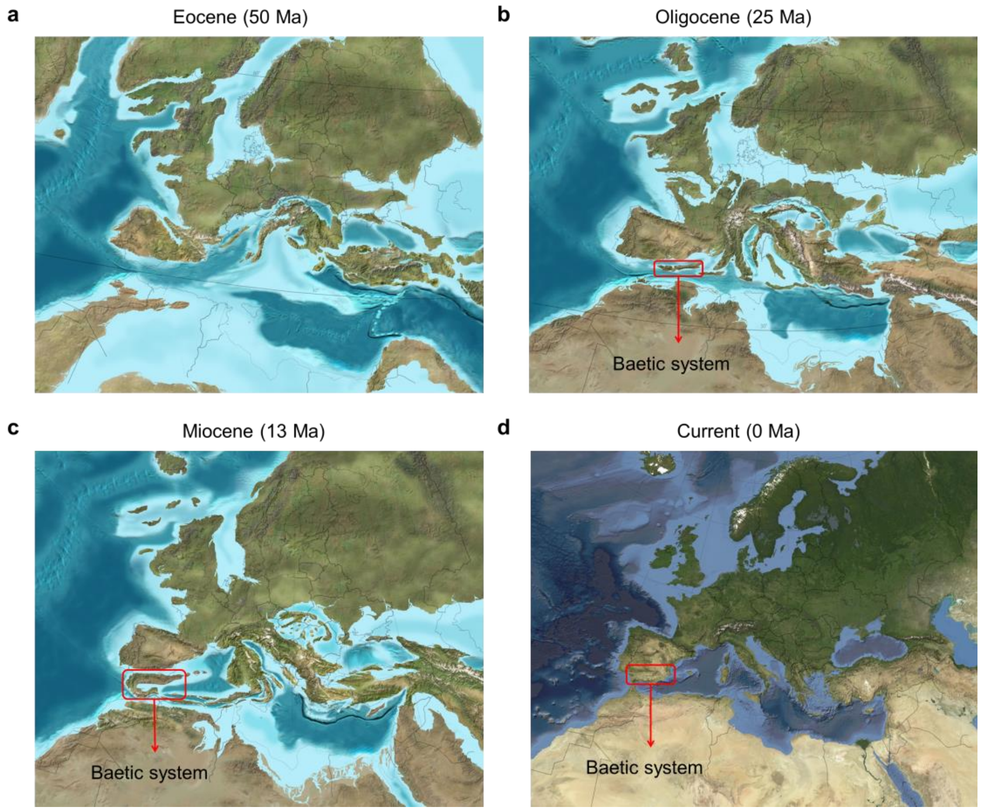

Fig. S5 Geographical patterns of the Mediterranean region in the last 50 million years. Ma refers to million years. The paleogeographical maps of North Africa, Europe, and the Mediterranean Sea were obtained from Colorado Plateau Geosystems, Inc. (http://cpgeosystems.com) (© Ron Blakey Colorado Plateau Geosystems Inc.).
